# College-aged women in the United States that play overhand throwing sports have masculine digit ratios

**DOI:** 10.1371/journal.pone.0203685

**Published:** 2018-09-13

**Authors:** Michael P. Lombardo, Sango Otieno, Adam Heiss

**Affiliations:** 1 Biology Department, Grand Valley State University, Allendale, Michigan, United States of America; 2 Statistics Department, Grand Valley State University, Allendale, Michigan, United States of America; University of Vienna, AUSTRIA

## Abstract

Athletic prowess in both males and females is negatively correlated with the ratio between the lengths of the second and fourth fingers (2D:4D), a correlate of prenatal testosterone exposure. Because multiple lines of evidence suggest that prenatal testosterone exposure is associated with sports interest, motivation, and athletic performance we measured the digit ratios of 77 non-athletes, 103 varsity athletes, and 78 club sport athletes to test 8 hypotheses about the relationship between digit ratio and the athletic behavior of college-age women in the USA. Using independent samples *t*-tests, we found no significant differences between the digit ratios of women that (1) were athletes and non-athletes, (2) were varsity or club sport athletes, (3) had played or were currently playing individual or team sports, (4) played contact and non-contact sports, (5) played sports involving a ball and those that do not, (6) played sports where the outcome was determined by a score or the outcome of direct physical competition or subjectively by judges, or (7) were starters or reserves on their teams. However, women that played overhand throwing sports softball and water polo had significantly smaller digit ratios than did women that played other sports. These differences were not due to scaling effects. The independent samples *t*-test results were supported by subsequent Monte Carlo bootstrap, Bayesian, Random Forest, and multiple linear regression analyses. We suggest that the organizational consequences of prenatal testosterone exposure may influence the anatomy and physiology of women that leads to success playing overhand throwing sports.

## Introduction

In humans, the ratio between finger 2 (2D) and finger 4 (4D), 2D:4D, is smaller in males than in females and is independent of age [1]. Experiments with rodents show that the length of 2D is positively influenced by prenatal exposure to estrogen whereas the length of 4D is positively influenced by prenatal testosterone exposure [2–4]. Evidence also suggests that prenatal testosterone exposure positively affects the length of finger 5 (5D) so that the 2D:5D ratios of males are also smaller, on average, than those of females. [5]. However, recent analyses suggest that the sexual dimorphism in 2D:4D may be due to a scaling effect with males exhibiting smaller 2D:4D because males are larger and have longer fingers, on average, than females [6–8]; but see [9].

In humans, 2D:4D is (a) established by the end of the first trimester [10] coinciding with a period of high testosterone production in male fetuses [11], (b) determined by the relative proportions of prenatal testosterone and estrogen early in development that affect digit growth during a relatively narrow window of time early in development [3], (c) relatively stable throughout postnatal growth [12–16], and (d) sexually dimorphic across ethnic groups both within and between countries [17,18].

Sex differences in athletic performance are well established. Postnatally, the greater exposure of males than females to testosterone beginning at the onset of puberty, when the differences between males and females in running speed and strength widen [19], is primarily responsible for these differences. Furthermore, the use of anabolic steroids to enhance athletic performance provides further evidence that androgens are major contributors to the sex differences in athletic performance [20,21]. Prenatal testosterone exposure may also affect subsequent athletic behavior and performance [22–24].

Athletic prowess in both males and females, as indicated by their performance on tests of physical skills [25–29] and level of athletic achievement [17,30–32], is associated with smaller 2D:4D [33,34]. The relationship between 2D:4D and athletic prowess may be mediated by the effects of prenatal testosterone exposure on the cardiovascular system [34]. The finding that the relationship between 2D:4D and athletic ability is especially strong for distance running [25,26,35–37] is consistent with this idea.

Multiple lines of evidence suggest that prenatal testosterone exposure influences not only sports performance but also sports interest and motivation [24,38]. First, the typical childhood play and activity patterns (e.g., rough-and-tumble play) of boys [39,40] are not only positively correlated with prenatal testosterone exposure but also predict their interest in sports as adults [41–43]. Second, prenatal testosterone exposure is associated with the sex differences in toy preferences, activity interests, and play patterns of children, including competitive sports [44]. Third, smaller 2D:4D is negatively correlated with participation in competitive sports [45]. Fourth, a twin study showed high heritability of 2D:4D perhaps helping to explain why parents that were superior athletes tended to have children with superior athletic prowess [46]. Last, females with Congenital Adrenal Hyperplasia (CAH), a condition caused by excessive prenatal exposure to androgens, are more likely than non-CAH females to display a strong interest in participating in stereotypically masculine sports (e.g., team sports, contact sports, and sports involving projectiles) [43,47,48]. Collectively, these studies challenge social constructivist theories [49–52] that hypothesize that males exhibit greater levels of interest and participation in sports than do females primarily because of sex-biased social and cultural influences such as differences in equality of opportunities and the socialization of children. Indeed, Deaner et al. [38] presented evidence that prenatal testosterone exposure was more important than socialization as a proximate factor influencing the well-established, cross-cultural sex differences in sports interest and participation [53–55].

Our purpose was to examine the relationship between digit ratio and the athletic behavior (e.g., participation in competitive sports and the sports played) and performance (e.g., level of competition attained) of women students and athletes at Grand Valley State University (GVSU). Compared to those for men, data on the relationship between digit ratio and athletic behavior and performance in women are relatively sparse [31,36,56–61]. One of our goals was to fill this gap in our knowledge by studying the relationship between digit ratio and the athletic behavior and performance of female college students currently playing competitive sports at GVSU. The results of previous research on the association between digit ratios and athletic prowess and physical fitness prompted us to test the following eight hypotheses.

### Hypothesis 1: Women varsity and club sport athletes have smaller digit ratios than do non-athletes

#### Background

We tested this hypothesis for several reasons. (a) A variety of studies have demonstrated negative correlations between 2D:4D and athletic prowess and participation in competitive sports [36,45,45]. (b) 2D:4D may be negatively correlated with temporary increases in circulating testosterone in men during “challenge” situations similar to those experienced during athletic competition [62] and greater sensitivity to levels of circulating testosterone in both men and women [63–65]. (c) Competitive social interactions are positively correlated with testosterone exposure [66,66–68]. (d) Polish women that play college sports tend to have higher levels of competitiveness than those that do not and the difference may be related to their greater prenatal testosterone exposure [31]. (e) CAH females are more likely than non-CAH females to show strong interest in stereotypically masculine sports [43,47,48]. (f) Male-typical childhood play and activity patterns, which are positively correlated with exposure to prenatal testosterone, predict adult sports interest [41,42].

### Hypothesis 2: Varsity athletes have smaller digit ratios than do club athletes

#### Background

We tested this hypothesis because varsity athletes are more highly valued by the University than club sport athletes because varsity athletes and their teams receive financial support from the University and, therefore, are likely to be superior athletes, on average, than club sport athletes.

### Hypothesis 3: Women that play team sports have smaller digit ratios than those that play individual sports

#### Background

We tested this hypothesis because boys have a greater propensity than girls to participate in physical competition and behaviors that require teamwork to confront a challenge [69,70] suggesting that prenatal testosterone exposure influences these behaviors. Several empirical studies support this hypothesis by demonstrating that pre-pubertal boys, with low levels of circulating testosterone, are more likely than girls to participate in both organized and spontaneous (e.g., “pick-up games”) team sports [55,71–73].

### Hypothesis 4: Women that play contact sports have smaller digit ratios than those that play non-contact sports

#### Background

We tested this hypothesis for several reasons. (a) Physical aggression is one manifestation of social competition [67,68] and contact sports require physical aggression from players as they try to displace competitors from preferred locations on the field as in “boxing out” in basketball, impede the progress of a competitor in rugby, or separate competitors from the ball as in soccer. (b) The physically competitive rough-and-tumble play displayed more often by boys than by girls [39,40] is positively correlated with prenatal testosterone exposure [41–43]. (c) In women, 2D:4D is negatively correlated with personality traits like aggressiveness, assertiveness, competitiveness, and dominance that would promote success in contact sports [74–77]. (d) Women that voluntarily chose to participate in the physically demanding combat sports of judo and boxing at a Polish military academy had smaller 2D:4D than those that chose aerobic exercise as their required athletic activity suggesting that the voluntary choice of a contact sport may be influenced by prenatal testosterone exposure [78]. (e) Male athletes that played contact sports had significantly smaller 2D:4D and higher levels of physical aggression than those that played noncontact sports [24]. (f) In general, sports with frequent contact between competitors entail greater risks of injury than do non-contact sports. Therefore, the preference for different sports that pose different risks of injury may reflect individual differences in risk-taking attitudes. Evidence suggests that individual differences in risk-taking attitudes may be influenced by differences in testosterone levels [67,79,80]. Several studies have demonstrated negative correlations between financial risk-taking and 2D:4D suggesting that prenatal testosterone exposure influences the risk-taking tendencies of adults [79–81].

### Hypothesis 5: Women that play sports involving balls, or other projectiles, have smaller digit ratios than those that play sports without projectiles

#### Background

We tested this hypothesis because the development of visuospatial ability, which would be a benefit while playing sports involving projectiles, have been hypothesized to be associated with prenatal testosterone exposure and thus 2D:4D [34].

### Hypothesis 6: Women that play sports that involve frequent overhand throwing have smaller digit ratios than those that do not

#### Background

We tested this hypothesis because several observations suggest that prenatal testosterone exposure, amongst other factors, may affect throwing biology. (a) In both traditional and modern societies, boys and men throw projectiles more often in combat, hunting, and sports than do girls and women [82–87]. (b) Boys and men typically throw faster, farther, and more accurately than do girls and women [19,88,89]. (c) Males outperform females at hitting a moving target with a thrown ball although the sex differences in throwing experience and physical strength affecting accuracy at hitting distant targets and may be partly responsible for some of this sex difference [90]. (d) Boys tend to achieve mature throwing actions earlier than do girls [91–94]. (e) Training does not eliminate the sex differences in throwing [95,96]. (f) Cultural influences have little effect on the sex differences in throwing [97,98].

### Hypothesis 7: Women that play sports where the outcomes of contests are determined by a score or the outcome of direct competition between competitors (e.g. running or swimming races) have smaller digit ratios than those that compete in sports (e.g., gymnastics) where the outcome is subjectively determined by judges

#### Background

We tested this hypothesis because “scored” sports involve direct physical competition between teams (e.g., soccer) or individuals (e.g., a swimming race). Boys and men tend to participate in sports that involve direct physical competition more often than do girls and women [38,53,55] suggesting a possible role for prenatal testosterone exposure influencing these behaviors.

### Hypothesis 8: Women that were starters on their athletic teams have smaller digit ratios than those that were reserves

#### Background

We tested this hypothesis because starters, on average, are likely to be superior athletes compared to reserves. For example, starters had smaller 2D:4D than did reserves on Australian women’s semi-professional basketball teams [56].

## Materials and methods

### Ethics statement

This project was approved by the Human Research Review Committee at GVSU (HRRC Project No. 16-084-H). Each subject provided written informed consent before participating in the study.

### Subject recruitment

We recruited subjects from the undergraduate female student population of GVSU’s enrollment of approximately 21,000 undergraduate students during the 2016–2017 academic year. We recruited subjects from Biology classes at GVSU by having their instructors announce the opportunity to participate in this project, by posting in buildings and athletic facilities on campus advertisement flyers describing the project, and by contacting varsity and club coaches and requesting their cooperation by allowing us to use email to recruit their team members as subjects. All of the varsity and club coaches and GVSU’s Head Athletic Trainer gave us permission to contact female student-athletes. We offered subjects a $10.00 gift card to a local retailer as an incentive to participate in this project.

Subjects came from three populations at GVSU; (a) female students that did not compete in intercollegiate athletics, but may have participated in sports in high school and or in intramural sports at GVSU, hereafter referred to as non-athletes, (b) varsity athletes that competed in intercollegiate athletics (basketball, cheerleading, cross country, golf, lacrosse, soccer, softball, swimming, tennis, track & field, volleyball) at the National Collegiate Athletic Association (NCAA) Division II level (http://www.ncaa.org), and (c) club athletes that competed in intercollegiate athletics (basketball, dance, ice hockey, lacrosse, rowing, rugby, soccer, softball, synchronized ice skating, volleyball, water polo) against club athletes from NCAA Division I, II, and III institutions. The NCAA Divisions reflect, on average, the financial commitments made by colleges and universities to their athletes. Division I institutions provide the most athletically related financial aid for student athletes, Division II institutions provide athletes limited financial aid, and Division III institutions do not provide athletically related financial aid (http://www.ncaa.org). Varsity athletic teams at GVSU are funded by the University whereas club athletic teams are funded by team members and donors. Cheerleading and dance teams were included as sports in some analyses because they participated in intercollegiate competitions. Both competitive cheerleading and dance require skills associated with athletic prowess including agility, flexibility, strength, and highly coordinated and synchronized actions with teammates. Cheerleading combined dance and gymnastics. The athletic prowess of the college athletes in our sample was likely to be superior to those of the non-athletes because both varsity and club women athletes at GVSU are among the best in Division II and have won numerous individual and team national championships (https://gvsulakers.com/, http://www.gvsuclubsports.com/).

### Measuring digit ratios

Lombardo measured the lengths of 2D, 4D, and 5D from digital images of both hands that were collected on an Epson^®^ V550 flatbed scanner and saved on a computer for analyses. Computer assisted measurements of digit lengths produce the most accurate and consistent digit measurements [99]. Before producing scans, we instructed subjects to remove rings and other jewelry that might affect digit measurements and to place both of their hands, palm down, on the scanner without pressing them on the scanner bed while images were produced so as to not distort the length of their fingers which can alter digit ratio measurements [100]. Although fingertip fat pads may have been differentially deformed when subjects placed their hands on the scanner, fingertip size is unrelated to image-based 2D:4D measurements [101]. The scanner produced black and white images with image resolution set at 400 dpi. A scale bar on each image was used to calibrate the ImageJ image analysis software (http://rsb.info.nih.gov/ij/) used to measure digit lengths from the mid-point of the finger crease proximal to the palm to the tip of the fleshy part of the finger. Each digit was measured twice, but not consecutively so that first measurements did not influence second measurements (see Statistical analyses). We used the mean of the two measurements to calculate 2D:4D and 2D:5D from each hand.

In some studies, right-side digit ratios appear to be more sensitive to prenatal exposure to sex steroids [17] and previous studies reported that 2D:4D sexual dimorphism is more pronounced on the right side [102–105]. However, we measured and report digit ratios from both hands because a meta-analysis of digit ratio data was inconclusive for right- or left-side bias and recommended the measuring for both right- and left-side digit ratios [45].

Digit 5 (5D) was measured on each hand because males have smaller 2D:5D than females suggesting that there is an underlying growth field across the fingers that is influenced by prenatal testosterone exposure [5,106].

### Coding athletic performance and behavior

Each subject completed a survey about their athletic behavior and performance after their hands were scanned. We defined athletic behavior in two ways, (a) whether a subject participated in competitive sports or not and (b) by the sport(s) played. We defined athletic performance as the level of competition achieved (e.g., high school, varsity sport, club sport, starter, reserve).

Non-athletes completed a survey that included the following questions:

1. Did you play organized sports in high school? We considered organized sports to include club (e.g., YMCA or YWCA) and school sports. Possible responses were yes and no.
2. If you played organized sports in high school, what sport(s) did you play? Possible responses were soccer, softball, basketball, swimming & diving, track & field (including cross country), volleyball, tennis, and other.
3. Were you a starter or reserve? Possible responses were starter and reserve. Varsity and club athletes completed a survey that included the following questions:
  1. What sport(s) do you play at the intercollegiate level? Respondents listed the sport(s) they played.
  2. Do you play a varsity or club sport? Possible responses were varsity and club.
  3. Are you a starter or a reserve? Possible responses were starter and reserve.

Both surveys included other questions, but the analyses of those data are not reported on here.

From their survey responses, we coded whether subjects played individual or team sports, contact or noncontact sports, ball or non-ball sports, sports that required frequent overhand throwing or not, sports where the outcome was determined subjectively by judges or by the outcome of direct competition between individuals or teams, and if they were in the lineup at the start of the competition or game (i.e., starter) or not (i.e., reserve).

We coded golf, swimming, tennis, and track & field as individual sports because their primary competitive context is one of individuals directly competing against one or more other competitors even though the cumulative outcomes of individual competitions (e.g., races) are used to determine a team’s success during intercollegiate competition. Cross-country runners were included in the track & field category because they all also competed in track & field. We coded basketball, cheerleading, dance, ice hockey, lacrosse, rowing, rugby, soccer, softball, swimming, synchronized skating, volleyball, and water polo as team sports. We coded as contact sports those in which physical contact between competitors commonly occurs and included basketball, ice hockey, rugby, soccer, and water polo. Body checking is not allowed in women’s ice hockey, but it is considered a body contact sport (http://www.usahockeyrulebook.com/). We coded noncontact sports those in which physical contact between competitors is uncommon or prohibited by the rules and included cheerleading, dance, golf, lacrosse, rowing, softball, swimming, tennis, track & field, synchronized skating, and volleyball. We coded lacrosse as a non-contact sport because of its restrictive rules, relative to ice hockey, governing physical contact between players on opposing teams (www.ncaapublications.com). Ball sports included basketball, golf, ice hockey, lacrosse, rugby, soccer, softball, tennis, volleyball, and water polo. Ice hockey was included in this category because the main objective of the game is to move a hockey puck (a hard flattened, rubber ball) into the opposing team’s goal. We coded all other sports as non-ball sports. We coded softball and water polo as sports that required frequent overhand throwing; all other sports were coded as non-overhand throwing sports. We coded basketball, ice hockey, golf, lacrosse, rowing, rugby, softball, swimming, tennis, track & field, volleyball, and water polo as “score” sports. Cheerleading, dance, and synchronized skating were coded as subjectively scored sports. We used survey responses to code subjects as either starters or reserves.

### Statistical analyses

We examined the data for normality using the Kolmogorov-Smirnov test and, where appropriate, used parametric and nonparametric statistical tests to determine if the data supported any of the hypotheses we tested.

We used one-way ANOVA to test the null hypothesis of no differences in the digit ratios of non-athletes, varsity athletes, and club athletes. We used independent samples *t*-tests to test the null hypotheses of no differences in the digit ratios of women that played either individual or team sports, contact or noncontact sports, ball or non-ball sports, overhand throwing or non-overhand throwing sports, scored or subjectively-scored sports, and were starters or reserves. We also compared the digit ratios of subjects that were of European and non-European ancestry because digit ratios may vary by ethnicity [17]. In all cases, Levene’s test for equality of variances showed that there were no significant differences between the compared groups in sample variances (all P > 0.05). However, to be statistically conservative and because in some cases sample sizes were widely disparate, we report adjusted Welch *t* values [107], df, and P values for the *t*-tests where equal sample variances were not assumed [108].

We further evaluated the statistical outcomes of the independent samples *t*-tests in several ways. First, we calculated the effect sizes (Cohen’s d) of all two sample comparisons; by convention, effect size of d = 0.8 is considered large, d = 0.5 medium, and d = 0.2 small [109]. Effect sizes were calculated using an effect size calculator found at www.uccs.edu/~lbecker/. Second, we calculated and report the results of sensitivity power analyses of all two-sample tests using the online program G*Power found at www.gpower.hhu.de/en.html. The sensitivity power analyses calculated the minimum detectable effect sizes given the sample sizes of each group in each independent samples *t*-test with α = 0.05 and 1-β = 0.80.

Third, we calculated Bayes factors to investigate whether the data supported the null or alternative hypotheses we tested [110,111]. We calculated Bayes factors for independent samples *t*-tests using the online Bayes factor calculator found at http://pcl.missouri.edu/bayesfactor. The calculator uses group sample sizes and *t*-values to calculate the Jeffreys-Zellner-Siow-Bayes (JZSB) factor and reports whether the data provide support for either the null or alternative hypotheses tested [110,111]. Last, we used Monte Carlo bootstrap methods [112] to examine the reliability of the outcomes of the independent samples *t*-tests that detected significantly smaller digit ratios in women that played sports that involved regular overhand throwing (see Results). Our procedure was as follows. First, right and left hand 2D:4D and 2D:5D were resampled with replacement 1000 times producing 1000 bootstrap samples. Each bootstrap sample, made up of 12 pairs of overhand throwing and non-overhand throwing subjects, each of sample size 17 (the number of subjects that played overhand throwing sports) was subjected to unpooled *t*-tests that tested the null hypothesis of no significant differences in the digit ratios between women that played sports that involved regular overhand throwing and those that did not. The proportion of *t*-tests that rejected the null hypothesis was recorded and the mean of the 1000 *t*-tests produced a bootstrap estimate of the proportion of statistically significant outcomes of the unpooled t-tests. Finally, this procedure was repeated 100 times to produce bootstrap 95% confidence limits around the mean number of times each null hypothesis was rejected for each digit ratio on each hand.

We used Random Forest analysis, a decision-tree based machine learning algorithm [113], to further evaluate the relationship between digit ratio and athletic behavior and performance. Briefly, Random Forest is a meta-learning algorithm which consists of many individual decision trees each of which “voted” on an overall classification for a given data set and chooses the individual classification with the most votes. Each decision tree was built from a random subset of the “training” dataset, using replacement, during this sampling. That is, some data were included more than once in the sample, and others were not. In building each decision tree, a model based on a different random subset of the training dataset and a random subset of the available variables was used to choose how best to partition the dataset at each node. The result of the analysis was a decision tree that represented the mode of the classification of the individual trees. Random Forest analyses reduce variance by averaging multiple decision trees that have sampled different parts of the same data set. In summary, the resulting decision tree models of the Random Forest represent the final ensemble model where each decision tree votes for the result and the majority wins. Random Forest analyses in this study produced trees for each digit ratio that indicated the categories of athletic behavior and performance most associated with differences between groups in their digit ratios.

We used the results of the Random Forest analysis to determine the variables to examine in a multiple linear regression analysis to further examine the relationship between digit ratios and athletic behavior and performance. Based on the results of the Random Forest analysis, we performed several multiple linear regression analyses by choosing either (a) overhand throwing, contact sports, college athlete as independent variables and right 2D:4D as the dependent variable, (b) overhand throwing, college athlete, and team sports and independent variables and right 2D:5D as the dependent variable, (c) overhand throwing, contact sport, college athlete, ball sports, and team sports as independent variables and left 2D:4D as the dependent variable, or (d) overhand throwing, contact sports, and college athlete as independent variables and left 2D:5D as the dependent variable.

Where appropriate, we used Holm-Bonferroni sequential corrections for multiple tests [114,115] using a calculator found at www.researchgate.net/publication/236969037_Holm-Bonferroni_Sequential_Correction_An_EXCEL_Calculator and report adjusted P values.

We used (a) SPSS 22.0 to perform one-way ANOVA, independent samples Welch’s *t*-tests, intraclass correlations [116], analysis of covariance (ANCOVA), and Kolmogorov-Smirnov tests [108], (b) SAS 9.4 to perform Monte Carlo boostrap analyses and multiple linear regressions [117], and (c) R to perform Random Forest analyses [118]. Data are reported as mean ± SD. All tests were two-tailed testing the null hypothesis of no statistical difference between compared groups. We considered differences between groups to be statistically significant if P ≤ 0.05. Except were otherwise indicated, the term “digit ratios” refer to the 2D:4D and 2D:5D on both hands.

## Results

We measured the hands of 258 women (77 non-athletes, 103 varsity athletes, 78 club athletes; Table 1). First and second measurements of all digits were highly correlated (all r > 0.970, all P < 0.001) and right- and left-hand digit lengths and ratios were significantly correlated (all P < 0.05). All intra-class correlation coefficients were greater than 0.970 (all P < 0.001) for digit lengths indicating that our measurements were reliable and measurement errors did not obscure differences in digit ratios between groups [116]. We did not detect significant differences between the digit ratios of women of European (n = 246) or non-European descent (n = 12) (all P ≥ 0.08) and the effect sizes in these comparisons were moderate (all d = 0.39–0.54) so we pooled all subjects together for subsequent analyses. Moreover, including or excluding women of non-European ancestry in our analyses would not have had an important effect on our overall results because 7/12 (58%) participated in college sports (basketball (n = 2), cheerleading (n = 1), soccer (n = 1), track and field/cross country (n = 3)), the remainder did not. Moreover, subjects of non-European ancestry made up only 7/181 (4%) of college athletes and 5/77(6%) of non-athletes.

**Table 1:** Digit ratios of female non-athletes, varsity athletes, and club athletes at Grand Valley State University.

				H_0_: Non-athletes = varsity athletes = club athletes		
Digit Ratio	Non-athletes	Varsity athletes	Club athletes	F	df	P
Right hand						
2D:4D	0.976 ± 0.041 (77)	0.973 ± 0.035 (103)	0.980 ± 0.029 (78)	0.82	2, 255	0.44
2D:5D	1.201 ± 0.068 (76)	1.205 ± 0.061 (102)	1.215 ± 0.045 (78)	1.41	2, 253	0.25
Left hand						
2D:4D	0.974 ± 0.037 (77)	0.970 ± 0.032 (103)	0.978 ± 0.030 (78)	1.35	2, 255	0.26
2D:5D	1.187 ± 0.058 (77)	1.120 ± 0.057 (103)	1.203 ± 0.054 (77)	1.52	2, 254	0.22

Digit ratios are reported as mean ± SD (n).

There were no statistically significant differences between non-athletes, varsity athletes, and club athletes in digit ratios on each hand (all P ≥ 0.22, Table 1). The effect sizes for all of these comparisons were negligible (all effect sizes, f ≤ 0.04). Moreover, there were no statistically significant differences between non-athletes and college athletes (i.e., varsity and club athletes combined) in their digit ratios on each hand (all P ≥ 0.12) or between varsity athletes and all other students combined (all P ≥ 0.15). Each effect size was small (all d ≤ 0.19) and each JZSB factor supported the null hypothesis of no difference between groups. Accordingly, all subjects were pooled together for subsequent analyses. Women college athletes, varsity and club combined, participated in the following sports; basketball (n = 14), cheerleading (n = 11), dance team (n = 8), golf (n = 5), ice hockey (n = 12), lacrosse (n = 15), rowing (n = 11), rugby (n = 16), soccer (n = 22), softball (n = 10), swimming (n = 8), synchronized skating (n = 4), tennis (n = 8), track & field/cross country (n = 24), volleyball (n = 12), and water polo (n = 1).

For women that played sports in high school or college, there were no statistically significant differences between women that played individual or team sports (all P ≥ 0.24), contact or non-contact sports (all P ≥ 0.16), sports that involved a ball or not (all P ≥ 0.06), sports where the outcome was determined by a score or the outcome of a race and those where the outcome was determined subjectively by judges (all P ≥ 0.28), or were starters or reserves on their respective athletic teams (all P ≥ 0.52) (Table 2). The effect sizes were small in each of these comparisons and smaller than the minimum detectable effect sizes (Table 2) indicating that our sample sizes were not large enough to detect significant differences between these groups. However, each JZSB factor supported the null hypothesis of no difference between groups (Table 2).

**Table 2:** Digit ratios and the athletic behavior of women students at Grand Valley State University.

Category of athletic behavior	Right 2D:4D	Right 2D:5D	Left 2D:4D	Left 2D:5D
Played individual sport	0.980 ± 0.035 (63)	1.210 ± 0.058 (63)	0.974 ± 0.031 (63)	1.194 ± 0.058 (63)
Played team sport	0.974 ± 0.036 (178)	1.204 ± 0.055 (176)	0.972 ± 0.057 (178)	1.195 ± 0.057 (177)
H_0_: Individual sport = team sport	*t*_111.88_ = 1.20	*t*_103.86_ = 0.72	*t*_115.22_ = 0.40	*t*_107.26_ = 0.05
	P = 0.23	P = 0.48	P = 0.69	P = 0.96
	d = 0.17	d = 0.11	d = 0.06	d = 0.02
	mdes = 0.53	mdes = 0.53	mdes = 0.53	mdes = 0.53
	JZSB = 3.22	JZSB = 4.93	JZSB = 5.83	JZSB = 6.27
	Null	Null	Null	Null
Played non-contact sport	0.978 ± 0.037 (160)	1.207 ± 0.056 (159)	0.973 ± 0.033 (160)	1.195 ± 0.059 (159)
Played contact sport	0.972 ± 0.034 (88)	1.203 ± 0.056 (87)	0.974 ± 0.032 (88)	1.195 ± 0.054 (88)
H_0_: Non-contact sport = Contact sport	*t*_192.66_ = 1.43	*t*_175.18_ = 0.48	*t*_186.85_ = 0.21	*t*_192.20_ = 0.13
	P = 0.15	P = 0.63	P = 0.84	P = 0.90
	d = 0.17	d = 0.07	d = 0.03	d = 0.00
	mdes = 0.48	mdes = 0.48	mdes = 0.48	mdes = 0.48
	JZSB = 2.64	JZSB = 6.16	JZSB = 6.76	JZSB = 6.84
	Null	Null	Null	Null
Played non-ball sport	0.979 ± 0.034 (90)	1.207 ± 0.054 (90)	0.979 ± 0.032 (90)	1.197 ± 0.058 (89)
Played ball sport	0.974 ± 0.037 (158)	1.205 ± 0.057 (156)	0.970 ± 0.033 (158)	1.193 ± 0.057 (158)
H_0_: Non-ball sport = Ball sport	*t*_197.97_ = 1.17	*t*_193.89_ = 0.22	*t*_189.50_ = 1.89	*t*_179.92_ = 0.51
	P = 0.25	P = 0.83	P = 0.06	P = 0.61
	d = 0.14	d = 0.04	d = 0.28	d = 0.07
	mdes = 0.48	mdes = 0.48	mdes = 0.48	mdes = 0.48
	JZSB = 3.66	JZSB = 6.76	JZSB = 1.29	JZSB = 6.11
	Null	Null	Null	Null
Played non-overhand throwing sport	0.978 ± 0.035 (241)	1.209 ± 0.056 (239)	0.975 ± 0.033 (241)	1.198 ± 0.056 (240)
Played overhand throwing sport	0.951 ± 0.035 (17)	1.172 ± 0.050 (17)	0.952 ± 0.029 (17)	1.157 ± 0.060 (17)
H_0_: Non-overhand throwing sport = Overhand throwing sport	*t*_18.36_ = 3.07	*t*_19.03_ = 2.70	*t*_18.59_ = 3.12	*t*_18.02_ = 2.75
	P = 0.006	P = 0.008	P = 0.006	P = 0.013
	d = 0.77	d = 0.70	d = 0.74	d = 0.71
	mdes = 0.71	mdes = 0.71	mdes = 0.71	mdes = 0.71
	JZSB = 14.25	JZSB = 5.69	JZSB = 16.28	JZSB = 6.39
	Alternative	Alternative	Alternative	Alternative
Scored sport	0.976 ± 0.036 (218)	1.206 ± 0.055 (216)	0.972 ± 0.035 (218)	1.192 ± 0.056 (217)
Judged sport	0.975 ± 0.037 (27)	1.202 ± 0.061 (27)	0.979 ± 0.035 (27)	1.206 ± 0.060 (27)
H_0_: Scored sport = Judged sport	*t*_32.34_ = 0.18	*t*_31.68_ = 0.27	*t*_31.66_ = 1.00	*t*_31.86_ = 1.10
	P = 0.86	P = 0.79	P = 0.32	P = 0.28
	d = 0.03	d = 0.07	d = 0.20	d = 0.03
	mdes = 0.57	mdes = 0.57	mdes = 0.57	mdes = 0.57
	JZSB = 4.59	JZSB = 4.51	JZSB = 3.00	JZSB = 2.73
	Null	Null	Null	Null
Starter on athletic team	0.977 ± 0.036 (192)	1.207 ± 0.056 (191)	0.973 ± 0.033 (192)	1.194 ± 0.054 (191)
Reserve on athletic team	0.973 ± 0.033 (51)	1.203 ± 0.033 (50)	0.974 ± 0.034 (51)	1.196 ± 0.065 (51)
H_0_: Starter = Reserve	*t*_84.53_ = 0.68	*t*_42.32_ = 0.39	*t*_75.53_ = 0.27	*t*_69.70_ = 0.14
	P = 0.52	P = 0.70	P = 0.78	P = 0.88
	d = 0.12	d = 0.07	d = 0.03	d = 0.03
	mdes = 0.44	mdes = 0.44	mdes = 0.44	mdes = 0.44
	JZSB = 4.75	JZSB = 5.44	JZSB = 5.69	JZSB = 5.82
	Null	Null	Null	Null

See text for definitions of different categories of athletic behavior. Digit ratios are reported as mean ± SD (n). *t*_df_ = Welch’s *t*. d = effect size. mdes = minimal detectable effect size given the reported sample sizes, α = 0.05, and 1-β = 0.80. JZSB = Jeffreys-Zellner-Siow Bayes factor that reports whether the data provide support for either the null or alternative hypotheses.

Women that played sports in high school (n = 6) or college (n = 11) that involved frequent overhand throwing (i.e., softball or water polo) had significantly smaller digit ratios than women that played sports either without overhand throwing or where overhand throwing is infrequent (e.g., basketball) (all P ≤ 0.013) (Table 2). The differences between the digit ratios of women that played overhand throwing sports and those that did not remained statistically significant when subjected to Holm-Bonferroni sequential corrections for multiple tests (all P_adjusted_ = 0.024). None of the women of non-European descent played an overhand throwing sport. For each digit ratio, the effect size was relatively large (d = 0.70–0.77) and the sensitivity power analyses revealed that the minimal detectable effect sizes were smaller than those detected for right and left 2D:4D, slightly greater than that detected for right 2D5D, and equal for left 2D5D indicating that, in general, our sample sizes were large enough to detect significant differences between the digit ratios of women that played overhand throwing sports and those that did not. Finally, the JZSB factors supported the alternative hypothesis that the digit ratios of women that played overhand throwing sports was not equal to those of women that played other sports (Table 2). Further support for these results comes from several other analyses.

**Fig 1:**
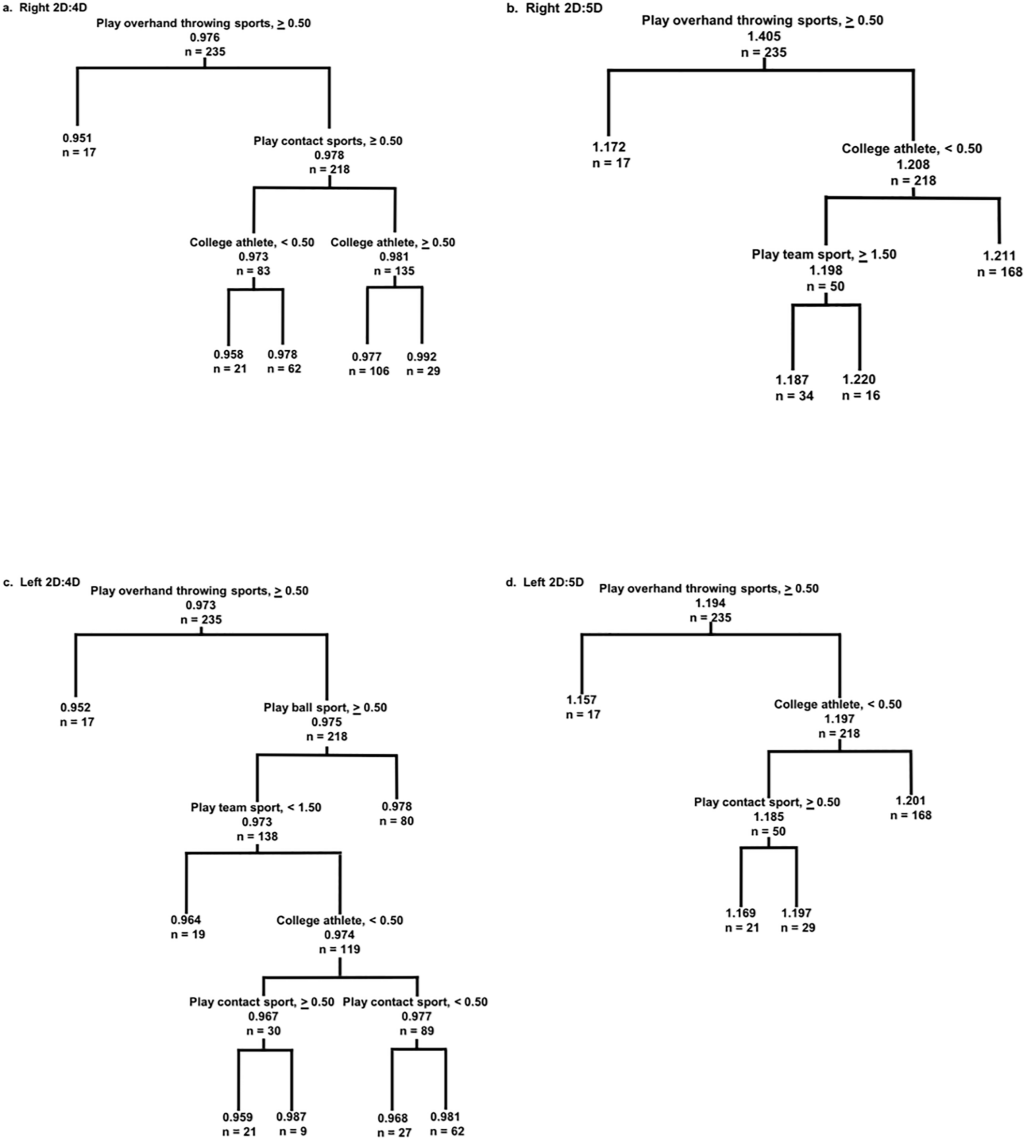
Random Forest regression trees predicting the digit ratios of women based on their athletic behavior. The regression trees illustrate the variables the Random Forest regression analyses identified to be important for sorting subjects by their digit ratios. The topmost node of each tree represents the most important explanatory variable sorting subjects by their digit ratios. For each digit ratio, the left-hand branch of the topmost node of each tree represents the 17 women that play or played sports (softball, water polo) that required frequent overhand throwing. Subsequent nodes represent the variables that significantly contributed to the model’s explanatory power. Terminal nodes suggest a natural clustering of homogenous groups. Values at each “leaf” represents the mean digit ratio of the observations (n) that were sorted by the regression analysis.

First, when we performed a Holm-Bonferroni sequential corrections for multiple tests, the differences between women that played overhand throwing sports and those that did not remained statistically significant for right 2D:4D (P_adjusted_ = 0.036), right 2D:5D (P_adjusted_ = 0.048), and left 2D:4D (P_adjusted_ = 0.036), but not for left 2D:5D (P_adjusted_ = 0.078). Second, the bootstrap analyses showed that the bootstrap 95% confidence intervals did not include 50% frequency of rejection of the null hypothesis for each digit ratio. The relationship between digit ratio and overhand throwing was strongest for right 2D:4D (left 2D:4D, 51.74–52.37%; left 2D:5D,56.45–57.08%; right 2D:4D, 61.87–62.43%; right 2D:5D, 50.05–50.68%). Second, the Random Forest analyses produced a decision tree for each digit ratio that indicated that the first decision node dividing the subjects into different groups was whether or not they played an overhand throwing sport (Fig 1). Other important branching nodes in the trees included (a) college athlete vs. non-college athlete (all digit ratios), contact vs. non-contact sports for right 2D:4D, left 2D:4D, and left 2D:5D, (b) individual vs. team sports for right 2D:5D and left 2D:4D, and (c) ball vs. non-ball sport for left 2D:4D (Fig 1). Last, multiple linear regression analyses revealed that whether or not a woman played an overhand throwing sport was the only variable that made a statistically significant contribution to models that statistically analyzed the relationships between digit ratios and the variables chosen by the Random Forest analyses as important categories of athletic behavior and performance (Table 3). However, after a Holm-Bonferroni sequential correction for multiple tests only right 2D:4D remained statistically significant (P_adjusted_ = 0.006) an outcome that is consistent with the results of the Monte Carlo Bootstrap analyses described above.

**Table 3:** Results of multiple linear regression analyses examining the relationships between digit ratios and the athletic behavior and performance of women student-athletes at Grand Valley State University.

	Right 2D:4D		Right 2D:5D		Left 2D:4D		Left 2D:5D	
	Wald χ^2^	P	Wald χ^2^	P	Wald χ^2^	P	Wald χ^2^	P
Overall model	9950.7	**< 0.001**	7477.5	**< 0.001**	10670.47	**< 0.001**	5882.5	**< 0.001**
Overhand throwing	10.47	**0.001**	5.51	**0.02**	4.87	**0.03**	7.34	**0.01**
Contact sport	3.08	0.08	--	--	0.59	0.44	0.21	0.86
College athlete	0.07	0.79	2.60	0.11	0.004	0.95	3.30	0.07
Ball sport	--	--	--	--	2.07	0.15	--	--
Team sport	--	--	0.19	0.66	0.09	0.76	--	--

The variables examined for each digit ratio in the regression analyses were chosen for inclusion in each model based on the variables determined by Random Forest analyses to be most associated with differences in digit ratios between subjects as classified by different categories of their athlete behavior (see Fig 1). Statistically significant associations are indicated with bold font. All df = 1.

Finally, we did not find evidence that the differences between the digit ratios of women that played overhand throwing sports and those that did not were due to scaling effects. First, women that playing throwing sports did not have statistically longer fingers than women who did not play throwing sports (Table 4). Second, when we performed four separate ANCOVAs setting for each the digit ratio on each hand as the dependent variable, whether women played a throwing sport or not as the fixed effect, and mean finger length on each hand as the covariate the statistical differences between the digit ratios of women that played throwing sports and those that did not remained. Moreover, the differences remained statistically significant after we performed a Holm-Bonferroni sequential correction for multiple tests (Table 5).

**Table 4:** Finger lengths of women students at Grand Valley State University.

Finger	Finger length of women that played overhand throwing sports	Finger length of women that played non-overhand throwing sports	Welch’s *t*	df	P
Right 2D	73.92 ± 5.72 (17)	73.92 ± 4.58 (241)	0.003	17.48	0.99
Right 4D	77.74 ± 5.41 (17)	75.64 ± 4.68 (241)	1.55	17.73	0.14
Right 5D	63.08 ± 4.02 (17)	61.22 ± 4.30 (239)	1.83	18.71	0.08
Left 2D	73.87 ± 5.14 (17)	73.58 ± 4.46 (241)	0.27	17.74	0.82
Left 4D	77.62 ± 5.25 (17)	75.51 ± 4.68 (241)	1.62	17.85	0.12
Left 5D	63.88 ± 4.61 (17)	61.49 ± 4.25 (240)	2.07	17.98	0.05

Finger lengths (mm) reported as means ± SD (n). The Welch *t*-test for left 5D became statistically insignificant after performing a Holm-Bonferroni sequential correction for multiple tests, P_adjusted_ = 0.30.

**Table 5:** Results of ANCOVAs comparing the digit ratios of women that played overhand playing sports with those that did not.

Digit Ratio	B ± SE	95% C.I.	Wald χ^2^	P	Model χ^2^	P
Right 2D4D	0.027 ± 0.009	0.010–0.044	9.45	0.002	9.28	0.010
Right 2D5D	0.036 ± 0.014	0.009–0.063	6.73	0.009	10.27	0.006
Left 2D4D	0.023 ± 0.008	0.007–0.093	7.67	0.006	8.48	0.014
Left 2D5D	0.038 ± 0.014	0.011–0.065	7.63	0.006	14.83	0.006

ANCOVAs was performed with digit ratios as the dependent variables, whether or not a women played an overhand throwing sport as a fixed effect and mean finger lengths as covariates. All Wald χ^2^ df = 1. All Wald χ^2^ P remained statistically significant after performing a Holm-Bonferroni sequential correction for multiple tests: Right 2D4D, P_adjusted_ = 0.008; Right 2D5D, P_adjusted_ = 0.018; Left 2D4D, P_adjusted_ = 0.018; Left 2D5D, P_adjusted_ = 0.018. All Model χ^2^ df = 2. All model χ^2^ P remained statistically significant after performing a Holm-Bonferroni sequential correction for multiple tests all P_adjusted_ = 0.024.

## Discussion

Only one of our eight hypotheses was supported: women who played sports that required frequent overhand throwing had statistically smaller digit ratios on each hand than those that played other sports. Our small sample of women that played overhand throwing sports requires that this result be verified by future studies. However, our confidence that our finding reflects a real difference between these groups in their prenatal testosterone exposure is reinforced for several reasons. First, we found significant differences between the groups for 2D:4D and 2D:5D on each hand, the effect sizes were large, the sensitivity power analyses revealed that our sample sizes, although unbalanced, were generally large enough to detect differences between the groups, the results did not change when we accounted for multiple tests using Holm-Bonferroni sequential corrections for multiple tests, and the JZSB factors supported the alternative hypothesis of a difference between groups for digit ratios on each hand. Second, the Random Forest analysis produced decision trees for each digit ratio that indicated that the first decision node dividing the subjects into different groups based on their digit ratios was whether or not they played an overhand throwing sport. Third, the multiple linear regression analyses revealed that whether or not a woman played an overhand throwing sport was the only variable that made a statistically significant contribution to the models that statistically analyzed the relationships between digit ratios and the variables chosen by the Random Forest analyses as important categories of athletic behavior and performance associated with the digit ratios of women student-athletes at GVSU. Last, the differences between the digit ratios of women that played overhand throwing sports and those that did not were not due to scaling effects: women that played throwing sports did not have longer fingers than those that did not.

We suggest two non-mutually exclusive hypotheses to explain why women that played overhand throwing sports had smaller digit ratios than those that did not. First, prenatal testosterone exposure may have resulted in an anatomy and physiology that led to success at playing overhand throwing sports. Second, prenatal testosterone exposure may have psychologically predisposed women to playing these kinds of sports. Historically, overhand throwing was an important component of male success in combat and hunting and may the at the root of the evolution of the well-established sex differences in throwing speed, distance, and accuracy [119] [119,120]. Therefore, it would be interesting to determine if males (a) that play overhand throwing sports have smaller digit ratios than those males that play sports that do not require overhand throwing and (b) with superior throwing ability (i.e., throw faster, farther, and more accurately) have smaller digit ratios than those with poorer throwing ability. The results of such a study would help reveal the relationship between prenatal testosterone exposure and throwing ability.

Our results indicate that digit ratios were not very accurate overall predictors of the athletic behavior and performance of the college-age women in this sample. We found no significant differences between athletes and non-athletes in digit ratios. This result is consistent with some studies [31,57,61,121] but not others [31,36,37,45,58,60,61,78,122–127]. These inconsistent findings suggest that prenatal testosterone exposure may have different effects on the development of traits associated with athletic prowess in males and females. For example, some studies show that smaller digit ratios are associated with superior performance at running long distances in both men and women [36,45] and may be associated with maximal oxygen uptake [35]. In contrast, the effects of prenatal testosterone exposure on adult hand grip strength are not consistent across the sexes with 2D:4D being negatively correlated with hand grip strength in college-aged men but not women [128].

We did not detect statistically significant relationships between digit ratios and whether women had played or were currently playing individual or team sports, contact sports, sports involving a ball, sports where the outcome was determined by a score or the outcome of direct physical competition or a subjectively by judges, or were starters or reserves on their teams. The differences between the digit ratios of the subjects in these categories of athletic behaviors were in the predicted direction in some cases but not others (Tables 1 and 2). However, for each comparison the minimal detectable effect sizes calculated by the sensitivity power analyses were greater than those we detected indicating that that our sample sizes were not large enough to detect statistically significant differences between these groups. However, the JZSB factors supported the null hypothesis of no difference between groups thereby contributing to our confidence in our interpretations. However, two predictors approached having significant effects on the multiple regression models in the direction of our predictions (Table 3). Consistent with our prediction, smaller right hand 2D:4D was associated with playing a contact sport and smaller left 2D:5D was associated with being a college athlete. Larger sample sizes could help clarify these relationships. Nevertheless, these results collectively suggest that prenatal testosterone exposure may have different organizational effects on the physiology and brains of developing females and males. Exposure to testosterone during development permanently affects the organization of the brain influencing subsequent patterns of behavior including, but not restricted to, aggression, gender orientation, interests, sexual orientation, and spatial abilities [44,129,130]. For example, we did not find a relationship between digit ratios and whether a woman played team or individual sports. In contrast, men at a Polish military academy that participated in individual sports (e.g., martial arts, running, swimming) had smaller digit ratios than men that participated in team sports (e.g., soccer, basketball, team handball, volleyball) [131], an outcome opposite of our prediction for women. The result of the study in the Polish military academy suggests that, at least for men, prenatal testosterone exposure may predispose men to participate in sports in which individual males compete directly with others. This hypothesis requires further testing.

The explanatory value of our study is possibly limited because we did not collect data on two variables that might have helped us clarify the relationship between digit ratios and the athletic behavior and performance of women students at GVSU. First, we did not record the dominant and non-dominant hands of our subjects. In one study, women college athletes had smaller digit ratios on their dominant hand than did non-athletes [58]. However, we do not think that data on the dominant hands of our subjects would have significantly altered our results because approximately 90% of the population is right-handed and left-handedness is less common in females than in males [132]. Therefore, it is likely that over 90% of the subjects in any of our sub-populations were right-handed. Given the very small effect sizes we detected in most comparisons between different categories of athletic behavior (Table 2), we think it unlikely that further subdividing our subject pool by analyzing subjects’ digit ratios by dominant and non-dominant hands would have revealed significant differences among the subpopulations.

Second, when we obtained images of the subjects’ hands we did not record the phase of their menstrual cycle if they happened to be normally cycling or, if they were not normally cycling and were using hormonal contraceptive, the type of hormonal contraceptive (e.g., monophasic or triphasic), and the day of oral contraceptive use. Menstrual cycling and hormonal contraceptive use and type may be confounding factors in the study of 2D:4D in premenopausal women because evidence suggests that in normally cycling women 2D:4D increases on the left, but not right, hand, during the pre-ovulatory (i.e., follicular) phase of the cycle and declines thereafter due to the effects of changing estrogen levels on bone and soft tissues [133]. In contrast, 2D:4D significantly changes on the right, but not left, hand across the 28-day course of hormonal contraceptive use [133]. However, we don’t think that this lack of data had a confounding effect on our results because most of the subjects were likely to be using hormonal contraceptives. The majority of female college athletes may use hormonal contraceptive pills to avoid menstruation during training or competition [134] and approximately 34% of college-aged women in the USA use hormonal contraceptive pills (~27%) or other kinds of hormonal contraception (~7%) [135]. Nevertheless, future studies could benefit by taking into account the hormonal state of a woman when calculating her digit ratio because of the subtle effects of changing circulating estrogen levels on digit length and symmetry [1,133,136]. Indeed, because of these effects, measures of 2D:4D in women may not be able to reliably detect differences between groups without controlling for the hormonal state of subjects [133]. Therefore, not controlling for the hormonal state of women subjects during digit ratio measurements may be the reason for inconsistent findings in the literature about the relationship between digit ratios and athletic behavior and performance in women.

Finally, our measurement methods may have influenced our results. We indirectly measured finger lengths using scans. A review of published digit ratio data showed that indirectly measuring 2D:4D tends to reduce the digit ratio with the effect being larger for females than for males [100]. However, we are confident that our measurement methods did not significantly affect our results because we (a) followed Ribeiro et al.’s recommendation and ensured that subjects lightly placed their hands on our scanner [100] and (b) made computer assisted measurements of digit lengths, the most accurate and consistent way to measure digits [99].

Despite these potential limitations, we detected a strong relationship between digit ratio and participation in overhand throwing sports further strengthening support for the hypothesis that prenatal testosterone exposure may predispose females for participation in sports that involve frequent overhand throwing. Because throwing is a skill historically related to the use of projectile weapons in combat and hunting [119,119] and is better developed in boys and men than in girls and women [19,89], our results suggest that prenatal testosterone exposure may influence the development of throwing in ability.

## Conclusions

Our results suggest that the relationships between digit ratio and athletic performance and behavior in women are complex and that prenatal testosterone exposure may have specific rather than general effects on the athletic ability of women. Some studies report significant associations between digit ratio and physical fitness and athletic achievement in women [31,36,37,45,56,58,60,61,78,122–127] while others do not [36,57,60,61,78,128,137–142]. Therefore, digit ratio alone may not be an accurate predictor of the effects of prenatal exposure of testosterone on the traits commonly associated with athletic behavior and performance in women and suggests that other, non-innate, factors (e.g., socialization) may also be important. Observations that the relationship between digit ratio and athletic prowess in men tends to be stronger than in women suggests that there has been stronger selection on males than females for the various testosterone-influenced traits that result in athletic prowess in the present. Testosterone-influenced traits associated with superior athletic prowess in the present are also associated with superiority in physical competition, combat, and hunting, endeavors that were and continue to be practiced more often by males than by females [53,143–146].
